# The accuracy of surrogate decision makers: informed consent in hypothetical acute stroke scenarios

**DOI:** 10.1186/1471-227X-13-18

**Published:** 2013-11-13

**Authors:** Jessica Bryant, Lesli E Skolarus, Barbara Smith, Eric E Adelman, William J Meurer

**Affiliations:** 1 University of Michigan, Medical School, Ann Arbor, MI 48109, USA; 2 Department of Neurology, University of Michigan, Ann Arbor, MI 48109, USA; 3 Department of Emergency Medicine, University of Michigan, Taubman Center B1354 SPC 5303, 1500 E. Medical Center Drive, Ann Arbor, MI 48109, USA

**Keywords:** Acute stroke, Cerebrovascular accident, Drug trials, Emergency medicine, Stroke care, Thrombolysis, TPA, Surrogate consent

## Abstract

**Background:**

Over one third of stroke patients have cognitive or language deficits such that they require surrogate consent for acute stroke treatment or enrollment into acute stroke trials. Little is known about the agreement of stroke patients and surrogates in this time-sensitive decision-making process. We sought to determine patient and surrogate agreement in 4 hypothetical acute stroke scenarios.

**Methods:**

We performed face to face interviews with ED patients at an academic teaching hospital from June to August 2011. Patients and the surrogates they designated were asked to make decisions regarding 4 hypothetical stroke scenarios: 2 were treatment decisions; 2 involved enrollment into a clinical trial. Percent agreement was calculated as measures of surrogate predictive ability.

**Results:**

A total of 200 patient/surrogate pairs were interviewed. Overall patient/surrogate percent agreement was 76.5%. Agreement for clinical scenarios ranged from 87% to 96% but dropped to 49%-74% for research scenarios.

**Conclusions:**

Surrogates accurately predict patient preferences for standard acute stroke treatments. However, the accuracy decreases when predicting research participation suggesting that the degree of surrogate agreement is dependent on the type of decision being made. Further research is needed to more thoroughly characterize surrogate decision-making in acute stroke situations.

## Background

By its nature, acute stroke treatment often involves cognitively-impaired-patients who need to make urgent decisions regarding high-risk-treatments within a short window of time. In fact, over 35% of stroke patients have acute cognitive or language difficulties that prohibit them from consenting to emergency acute stroke treatments [1]. Thus, acute stroke treatment decisions are often made by surrogate decision-makers (patient proxies), who are typically family members. Surrogate decision-making based on “substituted judgment”—the idea that surrogates choose the treatment the patient would most want to receive—is commonly employed in many fields of medicine to justify the treatment decisions that are made on behalf of incapacitated patients. Utilizing substituted judgment is a means by which medical professionals attempt to observe the ethical principle of respect for persons and patient autonomy in decision making.

Several studies have assessed the ability of surrogates to accurately predict patients’ preferences regarding both research participation [2,3] and the type of treatment in the critical care setting [4-8]. A systematic review revealed that surrogates predict ICU patients’ treatment preferences with only 68% accuracy [9]. Both patient designation of surrogates and prior surrogate-patient discussion of patients’ preferences failed to improve surrogates’ accuracy in predicting ICU patient desires [9]. Previous work on the accuracy of surrogates in predicting patients’ consent for research in the intensive care setting found false-positive (surrogate consenting when patient would not have) consent rates for both a low-risk and a high-risk study to be 16% and 20.3% respectively [3]. Other studies investigating surrogate ability to predict participation in clinical research scenarios found that surrogates frequently made decisions based on their own preferences and refused research participation by proxy when it was in fact desired [2,10]. Given significant observed discrepancies between the choices of surrogates and patients, controversy exists as to the importance, validity, and ethical integrity of obtaining surrogate consent for incapacitated patients [11-13].

Given the time-sensitive nature of the condition, surrogate consent for both treatment and research options on behalf of cognitively-impaired-patients with acute stroke is currently routine practice. The impact of surrogate consent within the acute stroke setting has not previously been investigated. We sought to determine the level of patient-surrogate agreement given hypothetical standard and experimental stroke treatment scenarios.

## Methods

### Setting

We performed cross-sectional surveys via face-to-face interviews of patients and their self-designated surrogates in the University of Michigan Hospital Emergency Department (ED) between June and August 2011.

### Participant identification

Patients were screened in the ED based on chief complaint and vital signs obtained from the ED electronic information system. Adult patients (ages 18 and over) were eligible for inclusion if they presented to the ED without a diagnosis of stroke or altered mental status, and with stable vital signs. In addition, participants had to be accompanied by a family member, friend, or significant other who might act as the surrogate decision maker in a real life setting.

### Scenarios

The scenarios were developed and then pilot-tested using semi-structured interviews in a small group of healthy, community dwelling individuals. Feedback regarding scenario wording and clarity informed adjustments to the scenarios and questions. Briefly, scenarios 1 and 2 asked the participants a treatment question only with no research element. Specifically, scenario 1 presented a choice between IV tPA and no treatment for stroke, while scenario 2 presented the treatment options of IV tPA or an endovascular clot removal procedure.

Scenarios 3 and 4 involved deciding between standard IV tPA and an experimental treatment within the context of a research trial. Scenario 4 was identical to scenario 3, except randomization in scenario 3 was fixed at 50:50 and response adaptive randomization was described for scenario 4. Response adaptive randomization changes the allocation ratio based on information accumulated during the trial to randomize more patients to whichever arm is performing better (possibly the active treatment or the control) [14]. The exact wording of the scenarios is available in Additional file 1.

### Interview procedures

After verbal informed consent was obtained, the surrogate was asked to leave the area. The patient was first asked if he/she could identify any of the warning signs of stroke, in order to assess his/her current knowledge of stroke symptoms [15]. Adequate stroke knowledge was defined as the ability to name two of five acute stroke symptoms (headache, paralysis, trouble speaking/confusion, vision changes, and dizziness). The patient was then presented with the four scenarios for decisions in the event of an acute stroke. The scenarios and data collection instrument are available as a web appendix.

After each scenario, the designated patient was asked to indicate his or her preferred treatment or research choice between the two offered. He/she was then presented with a 10-point Likert scale and asked to indicate his/her confidence in that decision—that he or she had made the right choice (0 = not at all confident, 10 = absolutely confident). The same scenarios were then presented to the surrogates, in the same order and with identical wording. Additionally, surrogates were asked to indicate their certainty that the patient made the same treatment or research choice. Again, a 10-point Likert scale was used for assessments (0 = completely unsure, 10 = completely sure). Patients and surrogates were allowed to ask questions regarding the scenarios before making decisions; the study team created a set of standard answers to the most commonly asked questions after the first week of recruitment. Demographics for patients/surrogates were collected after the completion of the interviews. No protected health information or specific patient or surrogate identifiers were collected. Zip codes were collected in order to estimate household incomes of the communities in which the patients and surrogates resided, using the census.gov website [16].

### Statistical analysis

The primary outcome variable was the overall weighted Cohen’s kappa statistic for agreement between patient and surrogate. This was calculated for each of the four scenarios. Other collected data, including demographic information and responses to the Likert scales for confidence and certainty of decisions made was summarized using means with standard deviation or proportions as appropriate. In addition, we also calculated Gwet’s AC1 which addresses the problem of a kappa statistic in low prevalence situations [17-19].

### Sample size calculation

We hypothesized that modest agreement would occur at baseline (kappa = 0.5) [16]. We assumed that patients would consent to hypothetical treatments or research trials 80% of the time, and that surrogates would consent 70% of the time. Based on our assumptions and using the N.cohen.kappa function from the Concord package of R Version 2.8.1, we estimated 90% power to detect a kappa statistic of 0.7 or higher with a total sample size of 200. We doubled this sample size to be conservative as we were aware of no prior pilot data that provided estimates of the performance of surrogate consent in acute stroke research.

### Human subjects protection

The study protocol was reviewed and approved by the University of Michigan Institutional Review Board and was granted Exempt status. Potential participants were provided with an information sheet describing the study and indicating that participation was completely voluntary. In addition, patients were assured that treatment for their presenting complaints would not be compromised by declining to participate. Participants were given the opportunity to have questions answered, and if they agreed, verbal consent was obtained.

## Results

### Subject characteristics

400 participants were enrolled in the study—200 “patients” and 200 “surrogates.” Characteristics of both are shown in Table 1. Stroke knowledge was similar between the patient and surrogate groups, with 71% and 74%, respectively, of patients and surrogates able to provide at least 2 out of 5 possible stroke symptoms. Mean community household income was the same for patients and surrogates at $54,144 and is higher than the national average of $41,994 [16].

**Table 1:** Patient and surrogate characteristics

	**Patients**		**Surrogates**	
**Characteristics**	**N**	**%**	**N**	**%**
Female	122	61%	120	60%
Male	78	39%	80	40%
Ethnicity				
White	178	89.5%	179	89.5%
African American	14	7%	14	7%
Hispanic	2	1%	2	1%
Asian	3	1%	2	1%
Other	3	1.5%	3	1.5%
Education				
Some high school	14	7%	8	4%
High school graduate	39	19.5%	44	22%
Some college	77	38.5%	61	30.5%
College graduate	44	22%	53	26.5%
Post graduate degree	26	13%	34	17%
Medical history				
Myocardial infarction	15	7.5%	5	2.5%
Diabetes	24	12%	15	7.5%
Stroke	6	3%	8	4%
Hypertension	49	24.5%	44	22%
Atrial fibrillation	14	7%	4	2%
None of the above	132	66%	144	72%
Surrogate relationship to patient				
Spouse			83	41.5%
Child			24	12%
Parent			29	14.5%
Sibling			10	5%
Significant other			21	10.5%
Other			28	14%
	**Mean**	**SD**	**Mean**	**SD**
Age, years	46.4	18.4	47.2	16
Number of siblings	2.5	1.8	2.4	1.9
Median household income	$54,144	$14,818	$54,144	$13,464

### Patient and surrogate decisions

Overall, surrogates predicted patients’ treatment preferences with 76.5% crude agreement; however, the kappa statistics for each scenario indicated poor agreement. The kappa statistics ranged from 0.18 in scenario 1 to -0.02 in scenario 4. The full results for each scenario are presented in Table 2. In scenario 1 in which subjects were given the choice between being given tPA or undergoing no treatment, surrogates had 96% prediction accuracy; the overwhelming majority of patients and surrogates chose the treatment of tPA over no treatment. Patient/surrogate agreement in scenario 2 (clot removal procedure vs. tPA) was 87%; the vast majority of agreement was found when both parties refused the procedure and opted for tPA instead.

**Table 2:** Agreement between patients and surrogates for hypothetical stroke scenarios

**Scenario 1: TPA vs. no treatment**				**Scenario 2: TPA vs. clot removal procedure**				**Scenario 3: TPA vs. standard RCT**				**Scenario 4: TPA vs. adaptive RCT**			
	**Patients**				**Patients**				**Patients**				**Patients**		
**Proxies**	Would you want TPA?	Yes	No	**Proxies**	Would you want the procedure?	Yes	No	**Proxies**	Would you want the trial?	Yes	No	**Proxies**	Would you want the trial?	Yes	No
	Yes	**95.5% (n = 191)**	2% (n = 4)		Yes	**1% (n = 2)**	4.5% (n = 9)		Yes	**4.5% (n = 9)**	9% (n = 18)		Yes	**28.5% (n = 57)**	21.5% (n = 43)
	No	2% (n = 4)	**0.5% (n = 1)**		No	8.5% (n = 17)	**86% (n = 172)**		No	17% (n = 34)	**69.5% (n = 139)**		No	29.5% (n = 59)	**20.5% (n = 41)**
		Mean	SD			Mean	SD			Mean	SD			Mean	SD
Patient confidence		8.0	2.1	Patient confidence		8.1	2	Patient confidence		8.0	1.9	Patient confidence		7.8	1.9
Proxy confidence		8.5	1.7	Proxy confidence		8.5	1.9	Proxy confidence		8.4	1.9	Proxy confidence		8.0	1.9
Proxy certainty		8.4	1.9	Proxy certainty		8.0	2.2	Proxy certainty		7.8	2.3	Proxy certainty		7.5	2.1
Cohen’s Kappa statistic		0.18		Cohen’s Kappa statistic		0.07		Cohen’s Kappa statistic		0.11		Cohen’s Kappa statistic		-0.02	
AC1 (95% CI)		0.96 (0.93-0.99)		AC1 (95% CI)		0.87 (0.79-0.91)		AC1 (95% CI)		0.63 (0.53-0.74)		AC1 (95% CI)		-0.01 (-0.13 – 0.13)	

Overall agreement is observed in the bolded diagonal cells (i.e for scenario 1: 96% of patients and proxies agreed). Counts and proportions listed for all scenarios. Patient and surrogate confidence was measured on a 10 point Likert scale. SD = standard deviation. tPA = tissue plasminogen activator. RCT = Randomized controlled trial. AC1 = Gwet’s agreement coefficient. CI = confidence interval.

In scenario 3 (tPA vs. standard RCT), 5% of patient/surrogate pairs consented to the trial while 70% of pairs refused the trial, resulting in an agreement rate of 74%. The majority of disagreement (65%) was found when patients desired the trial but proxies refused. Lastly, scenario 4 (tPA vs. adaptive RCT) represented the lowest rate of surrogate/proxy agreement at 49%, (pairs consenting to trial 28.5%, pairs refusing trial 20.5%) although there was higher overall consent to the trial in patients (56%) and proxies (50%) when compared to the standard RCT. Sensitivity and specificity were both 49%.

Patients and surrogates all indicated relatively high degrees of confidence (mean confidence scores for each scenario ranged from 7.8 to 8.5 on a 10-point Likert scale) in the decisions they were making. In addition, surrogates indicated that they were reasonably certain (mean certainty ranged from 7.5 to 8.4) that their decisions would be in concordance with the patients’ wishes.

## Discussion and conclusions

Our study of 200 patient/surrogate pairs found substantially more patient/surrogate agreement in scenarios involving standard treatments (scenarios 1 and 2) than for research protocols (scenarios 3 and 4). The varying predictive accuracy of surrogate decision-makers demonstrated in other studies raises questions about the ethics and validity of charging surrogates with important medical decisions, especially in an acute setting such as stroke [4,11]. Some ethicists argue against the use of surrogates as the decisional authority for incapacitated patients and recommend instead that surrogates serve as advisors to the medical team, who will carry out “best-interests judgment” on behalf of the patient; this is less clear regarding research participation [13]. The results of the current investigation suggest surrogates predict patient preferences for standard treatments well and perform less well when trying to predict patient preferences for research participation. It is likely that the degree of surrogate agreement is highly dependent on the type of decision being made.

When given a choice, the majority of patients and surrogates opted for standard treatments (tPA) over more experimental (scenario 2) or clinical trial (scenarios 3 and 4) alternatives. Possible explanations for this result could be risk aversion given the severe nature of the described stroke and/or time pressure to make potentially life and death decisions, leading participants to “stick with the standard” rather than choosing a newer, riskier, and often more complex alternative that would have required more thought [2,10]. Furthermore, the nature of our interview study limited the amount of information we could give participants regarding treatments and trials; participants may have opted for more standard treatments simply because they felt that not enough information was provided for the alternative research treatments. Our results echo the findings of several other studies in that surrogates predicted more frequently on the conservative side, (e.g., refusing research trial participation) when the patients themselves would have chosen (potentially riskier) research over standard treatment [2,10].

The clinical trial featuring response adaptive randomization (scenario 4) was more often agreed to by both patients and surrogates than the standard clinical trial (scenario 3), although there was substantially more disagreement regarding participation in the adaptive trial. It is possible that the complexity of the adaptive trial, confusion regarding its setup, and difficulty explaining it could have contributed to the observed discrepancy. Interestingly, scenario 4 represented the lowest rate of patient/surrogate agreement and also represented the lowest confidence in decision ratings from both patients and proxies. Proxy certainty that their decision was in accordance with the patient’s decision was also lowest in scenario 4 in comparison to the other scenarios. This suggests that patients and proxies are to some degree calibrated regarding the validity of their decisions, and that lack of proxy certainty is correlated with poor predictive accuracy. The therapeutic misconception (potential research volunteer belief that a research protocol confers medical benefit) has previously been described as a concern that response adaptive randomization may help mitigate [20]; we did observe numerically higher participation in the RAR scenario versus the fixed randomization design.

We recognize several limitations of our study. The precision of our estimates of agreement via the kappa statistic was limited, mainly because of the high prevalence of acceptance of standard treatments and the low prevalence of acceptance of research protocols by both patients and surrogates. This phenomenon is known as the “kappa paradox” and occurs in settings where the prevalence of a “yes” response is very high (i.e., consenting to the standard treatment of tPA) or very low (i.e., refusing participation in the research trials) [21]. The high or low prevalence of “yes” responses results in a high likelihood that agreement between surrogates and patients occurred by chance alone—a point that when taken into account by kappa results in low kappa statistics despite high rates of agreement between groups. For this reason, the Cohen kappa statistics here do not adequately reflect the degree of agreement between patients and surrogate decision-makers. Our calculations of the AC1 may provide a more meaningful picture, indicating fairly good to excellent agreement on scenarios 1 to 3. Another limitation is that our secondary measurements of certainty and confidence were on the Likert scale, and required that participants make probability estimations by choosing from ordinal numbers from 0 to 10. An additional limitation is that hypothetical scenarios raised many questions from subjects that likely caused participants to receive more, less, or different information than others. Although we developed a set of answers to commonly asked questions early in the study in an effort to standardize information as much as possible, it is not possible to anticipate every question for every discrete treatment or trial. In fact, this reflects the “real world” consent process. Furthermore, participants were not provided with an option for “undecided.” This undoubtedly forced participants into decisions they were not comfortable with or did not fully understand. However, this is identical to what happens in the setting of actual acute stroke. Finally, since these scenarios were hypothetical, it is unclear how our research would compare to patient/surrogate decisions in actual acute stroke presentations.

As to the generalizability of this study, the population was recruited from the area surrounding the University of Michigan Medical Center, and was therefore on average more educated, predominately Caucasian, and lived in areas of higher median income than the overall US population. Thus, the results of this study may not be generalizable to other, more urban or rural medical centers. Furthermore, patient/surrogate pairs were younger than typical stroke patient/surrogate pairs, potentially leading to results that do not reflect the preferences of the actual stroke victims and their surrogates in clinical practice. This is important to consider as the young may discount the quality of life of older adults – therefore adult children may act differently as surrogates decision makers compared to spouses/significant others.

In summary, patient/surrogate agreement varies depending on the type of clinical or research decision being made. Future research might investigate how patient/surrogate agreement compares to patient/physician agreement as medical providers are the most likely alternative decision makers when surrogates cannot be identified. An additional important area for future research is the degree to which potential research subjects understand the trial designs. Further research designs may benefit from a mixed methods approach to explore in more detail the nature of patient/surrogate discrepancies in order to develop a conceptual model for surrogate decision-making in acute settings.

## Competing interests

The authors declare that they have no competing interests.

## Authors’ contributions

JB was responsible for acquisition of the data, interpretation of the data, drafting of the manuscript, and administrative, technical and material support. WM provided study concept and design, analysis and interpretation of the data, revision of the manuscript, and study supervision. LS and EA aided in interpretation of the data and manuscript revision. BS provided administrative, technical and material support. All authors read and approved the final manuscript.

## Pre-publication history

The pre-publication history for this paper can be accessed here:

http://www.biomedcentral.com/1471-227X/13/18/prepub

## Supplementary Material

Additional file 1 Full text of hypothetical scenarios presented to research subjects. Click here for file
