# Supplementary material for: The accuracy of surrogate decision makers: informed consent in hypothetical acute stroke scenarios
Source: BMC Emerg Med. 2013 Nov 13;13:18. doi: 10.1186/1471-227X-13-18 (PMC4225766; doi:10.1186/1471-227X-13-18)
Supplement: Additional file 1 — Full text of hypothetical scenarios presented to research subjects. [file 1471-227X-13-18-S1.docx]

**Scenarios**

These four scenarios will be given the same order.

**Overview**

You (or your significant other/family member) have suffered a stroke. Without warning, you are unable to move the right side of your body (arm or leg) and are unable to talk. You are also unable to understand what others are saying. You have been taken to the nearest emergency department and doctors have done tests and determined that this condition has been caused by a clot in one of the blood vessels in your brain. We will now describe several possible treatment options. For each situation, you will choose between either the treatment and an alternative treatment OR the treatment and NO TREATMENT at all.

**Scenario 1**

You are a candidate for a drug called tissue plasminogen activator (tpa). This drug tpa has been+ FDA approved to reduce disability following stroke since 1996 and has been used extensively. It works by dissolving clots in the blood vessels of the brain. The original trial was funded by the U.S. government. It is recommended by the American Heart Association. For every 100 patients treated, about 13 extra patients would be left with no disability at 3 months when this drug is compared to receiving no acute treatment. By no disability we mean you would be able to walk on your own, care for yourself and return to work or other leisure activities that you enjoy. There is a small risk of serious bleeding with this drug (about 6 out of 100 patients). There is no difference in the chance of dying whether you receive this drug or not.

Treatment to agree to: tPA (versus nothing)

**Scenario 2**

You may choose to either receive a drug called tissue plasminogen activator (tpa) or a clot removal procedure. This drug tpa has been FDA approved to reduce disability following stroke since 1996 and has been used extensively. It works by dissolving clots in the blood vessels of the brain. The original trial was funded by the U.S. government. It is recommended by the American Heart Association. For every 100 patients treated, about 13 extra patients would be left with no disability at 3 months when this drug is compared to receiving no acute treatment. By no disability we mean you would be able to walk on your own, care for yourself and return to work or other leisure activities that you enjoy. There is a small risk of serious bleeding with this drug (about 6 out of 100 patients). There is no difference in the chance of dying whether you receive this drug or not.

The clot remover is an FDA approved device. It has been studied in trials funded by the device manufacturers. It has not been directly compared to tPA and trials only evaluated the safety of the device and its ability to remove clot. The ability to reduce disability at 3 months by this type of device is not currently known, but it is believed that when the device successfully removes the clot patients have a better chance at a full recovery. There is a small risk of serious bleeding or damage to the brain blood vessels with this device (about 6-10 out of 100 patients).

Treatment to agree to: clot removal (versus tpa).

**Scenario 3**

You may choose to either receive a drug called tissue plasminogen activator (tpa) or an experimental drug called experimental plasminogen activator (xpa). This drug tpa has been FDA approved to reduce disability following stroke since 1996 and has been used extensively. It works by dissolving clots in the blood vessels of the brain. The original trial was funded by the U.S. government. It is recommended by the American Heart Association. For every 100 patients treated, about 13 extra patients would be left with no disability at 3 months when this drug is compared to receiving no acute treatment. By no disability we mean you would be able to walk on your own, care for yourself and return to work or other leisure activities that you enjoy. There is a small risk of serious bleeding with this drug (about 6 out of 100 patients). There is no difference in the chance of dying whether you receive this drug or not.

The experimental drug xpa has been used extensively for patients with heart attacks and is now being investigated for stroke. This trial is being funded by the U.S. government.  The study is designed to answer the question of whether xpa is potentially better and safer than the current standard treatment tpa. The reason we are doing the trial is because we are truly uncertain whether xpa is better than tpa. In this trial, you will have a 50:50 chance of either receiving tpa (the standard treatment) or xpa (the new treatment).

Treatment to agree to: standard RCT with xpa (new treatment) versus tpa

**Scenario 4**

You may choose to either receive a drug called tissue plasminogen activator (tpa) or an experimental drug called experimental plasminogen activator (xpa). This drug tpa has been FDA approved to reduce disability following stroke since 1996 and has been used extensively. It works by dissolving clots in the blood vessels of the brain. The original trial was funded by the U.S. government. It is recommended by the American Heart Association. For every 100 patients treated, about 13 extra patients would be left with no disability at 3 months when this drug is compared to receiving no acute treatment. By no disability we mean you would be able to walk on your own, care for yourself and return to work or other leisure activities that you enjoy. There is a small risk of serious bleeding with this drug (about 6 out of 100 patients). There is no difference in the chance of dying whether you receive this drug or not.

The experimental drug xpa has been used extensively for patients with heart attacks and is now being investigated for stroke. This trial is being funded by the U.S. government. The study is designed to answer the question of whether xpa is potentially better and safer than the current standard treatment tpa. The reason we are doing the trial is because we are truly uncertain whether xpa is better than tpa. In this trial, the study will decide which treatment you will receive. Using data from patients already enrolled in the trial, you will have an increased chance of receiving whichever treatment (the standard tpa or the new treatment xpa) is better able reduce disability in other patients like you.

Treatment to agree to: adaptive RCT with xpa (new treatment) versus tpa
